# Case report: Expansion of phenotypic and genotypic data in *TENM3*-related syndrome: Report of two cases

**DOI:** 10.3389/fped.2023.1111771

**Published:** 2023-02-24

**Authors:** Fen Lu, Xin Xu, Bixia Zheng, Chunli Wang, Wei Zhou, Jian Tang, Xiaoke Zhao

**Affiliations:** ^1^ Department of Rehabilitation, Children's Hospital of Nanjing Medical University, Nanjing, China; ^2^ Nanjing Key Laboratory of Pediatrics, Children's Hospital of Nanjing Medical University, Nanjing, China

**Keywords:** syndromic microphthalmia, *TENM3*, whole exome sequencing, genotype-phenotype, children

## Abstract

Biallelic *TENM3* variants were recently reported to cause non-syndromic microphthalmia with coloboma-9 (MCOPCB9) and microphthalmia and/or coloboma with developmental delay (MCOPS15). To date, only eight syndromic and non-syndromic microphthalmia cases with recessive *TENM3* variants have been reported. Herein, we report two unrelated new cases with biallelic variants in *TENM3*, widening the molecular and clinical spectrum. Regarding patient 1, WES revealed compound heterozygous variants in the *TENM3* gene: c.3847_3855del; p.Leu1283_Ser1285del and c.3698_3699insA; p.Thr1233Thrfs*20 in the index patient, who was presenting with bilateral microphthalmia, congenital cataract, microcephaly, and global developmental delay. Regarding patient 2, compound missense heterozygous variants in the *TENM3* gene were identified: c.941C > T; p.Ala314Val and c.6464T > C; p.Leu2155Pro in the 3-year-old boy, who presented with congenital esotropia, speech delay, and motor developmental delay. The clinical features of these two cases revealed high concordance with the previously reported cases, including microphthalmia and developmental delay. The presence of microcephaly in our patient potentially expands the neurologic phenotype associated with loss of function variants in *TENM3*, as microcephaly has not previously been described. Furthermore, we present evidence that missense variants in *TENM3* are associated with similar, but milder, ocular features.

## Introduction

Teneurin transmembrane protein 3 (TENM3) encodes a large transmembrane protein involved in neural development by regulating the establishment of proper connectivity within the nervous system (1–3). It has been found to play a role in the development of the human eye by regulating the formation of ipsilateral retinal mapping to both the dorsal lateral geniculate nucleus and the superior colliculus (4–6). The homozygous null variant was first reported in a Saudi Arabian consanguineous family with non-syndromic bilateral colobomatous microphthalmia (7). Subsequently, very few publications have reported patients with *TENM3* variant-related syndromic microphthalmia to date (8–10). Here, we present two patients with recessive variants in *TENM3*, and we describe their clinical presentations, providing further clinical and molecular delineation of the *TENM3* syndrome.

## Materials and methods

### Study participants

Following informed consent, we obtained pedigree information, clinical data, and blood samples from the families. We obtained approval for human subject research from the ethics committee of the Children's Hospital of Nanjing Medical University.

### Whole exome sequencing

Trio-based WES was performed as previously described (11). In brief, genomic DNA was isolated from blood lymphocytes using the DNA isolation kit (Tiangen, China). Genomic DNA was sheared into fragments and then hybridized with the xGen Exome Research Panel v1.0 probe sequence capture array from IDT (Integrated Device Technology, United States) to enrich the exonic region. The enriched libraries were analyzed on an Illumina HiSeq XTen (Illumina, United States) platform. Low-quality variations of the quality score <20 (Q20) were filtered out. Sequencing reads were mapped to the GRCh37/Hg19 reference genome *via* Burrows-Wheeler Aligner (BWA) software. Single nucleotide variation (SNV) and inserts and deletions (INDEL) were filtered using GATK software (https://software.broadinstitute.org/gatk/). All identified variants were filtered using the 1000 Genomes Project (Chinese), dbSNP, Genome Aggregation Database (gnomAD), and ExAC database. Variants with a minor allele frequency higher than 5% were filtered out. Finally, the candidate variants were evaluated using the ACMG (American College of Medical Genetics and Genomics) criteria and further validated by direct Sanger sequencing.

### TA cloning of mutant PCR products

The two heterozygous *TENM3* variants in family 1 were both located in exon 19. To obtain a clean Sanger sequence of the two heterozygous variants, we cloned 383 bp-long PCR products of *TENM3* exon19 using the pCR2.1-TOPO plasmid vector system (Invitrogen). PCR products were generated using *TENM3* forward primer 5-ATCCTCAGCGTCAGGCAAGGAA-3 and reverse primer 5-TCCCCTGTCCCTGCGACGAC-3. The TA clone sequencing was conducted as previously described (12).

### Consideration of structural data and evolutionary conservation for variant evaluation

Protein domain structure depictions and evaluation were based on the UniProt (Universal Protein Resource) database. Orthologous proteins used to evaluate evolutionary conservation were obtained from the Ensemble Genome Browser and were aligned using the Clustal Omega multiple sequence alignment tool (EMBL-EBI). And we evaluated the crystal structure of the two missense *TENM3* variants in patient 2 using the online server, UCSF ChimeraX (http://www.cgl.ucsf.edu/chimerax//).

## Results

### Clinical findings

**Figure 1:**
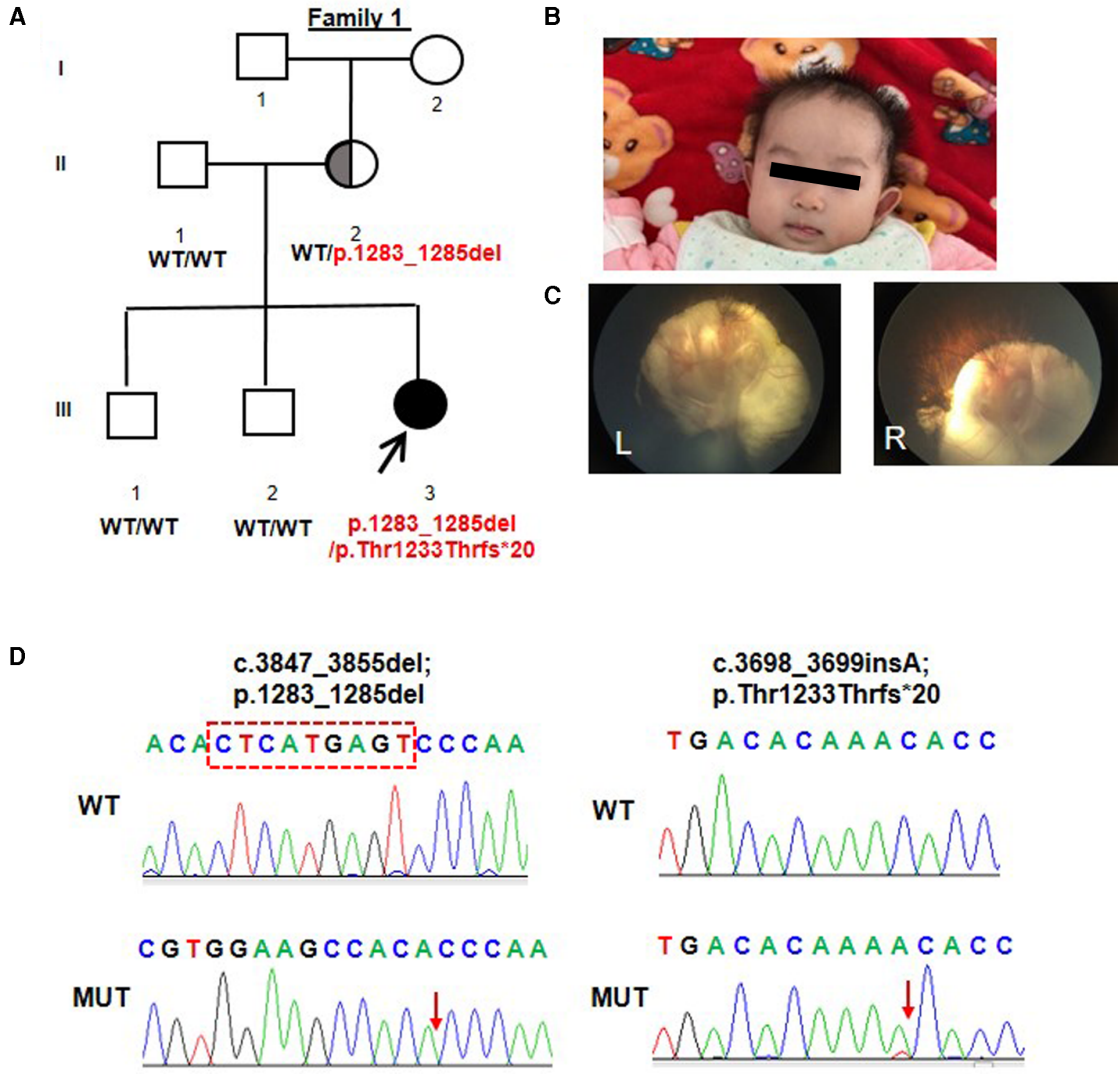
Trio-based WES identified compound heterozygous variants (p.Leu1283_Ser1285del; p.Thr1233Thrfs*20) in *TENM3* in a patient with bilateral microphthalmia, global developmental delay, and microcephaly. (**A**) Pedigree and genotype information for members of family 1. Squares indicate males, circles indicate females, filled symbols indicate affected individuals, and open symbols indicate healthy individuals. Patient 1 is denoted by a black arrow. The proband carried compound heterozygous variants: c.3847_3855del; p.Leu1283_Ser1285del and c.3698_3699insA; p.Thr1233Thrfs*20. The mother (II-2) with heterozygosity of the p.Thr1233Thrfs*20 variant had left exophthalmia and graduated from middle school with poor grades. (**B**) Facial picture of the proband at the age of 5 months with microphthalmia, prominent and low-set ears, and microcephaly. (**C**) Fundus examination of patient 1 revealed the posterior pole of the retina colobomas involving the optic discs and the fovea. (**D**) A TA clone sequencing from the genomic DNA of patient 1 including the fragment of exon 19 showed the two variants: c.3847_3855del; p.Leu1283_Ser1285del and c.3698_3699insA; p.Thr1233Thrfs*20. WT, wild type; MUT, mutant type.

Patient 1 was the 5-month-old daughter of Chinese non-consanguineous parents. She had two older brothers and both are healthy. She was born at 39 weeks of gestation. Her birth weight was 2.7 kg (−1.46 SD) and her length was 40 cm (−5.89 SD). At birth, she was diagnosed with bilateral microphthalmia and congenital cataract and she appeared to have pendular nystagmus and esotropia (Figure 1B). She first visited our department of rehabilitation at the age of 5 months for developmental delay. Her developmental milestones were delayed. Her head control was unstable and she was unable to turn over and grab the toy on her chest. On careful physical examination, her height, weight, and head circumference were 57 cm (−4.03 SD), 5 kg (−3.75 SD), and 37 cm (−4.28 SD), respectively. Her prominent and low-set ears were noted (Figure 1B). Fundus examination revealed the posterior pole of the retina colobomas involving the optic discs and the fovea (Figure 1C). Her hearing assessment was normal. According to the Gesell Developmental Diagnostic Scale for children, the proband's gross motor skills indicated a developmental age of 8 weeks; the fine motor skills indicated a developmental age of 8 weeks; Her blood counts, liver and renal function tests, thyroid profile, and metabolic screen by mass spectrometry were normal. Brain magnetic resonance imaging (MRI) showed no structure malformations except a widening in the frontotemporal extracerebral space. Her mother had left exotropia and graduated from middle school with poor grades.

**Figure 2:**
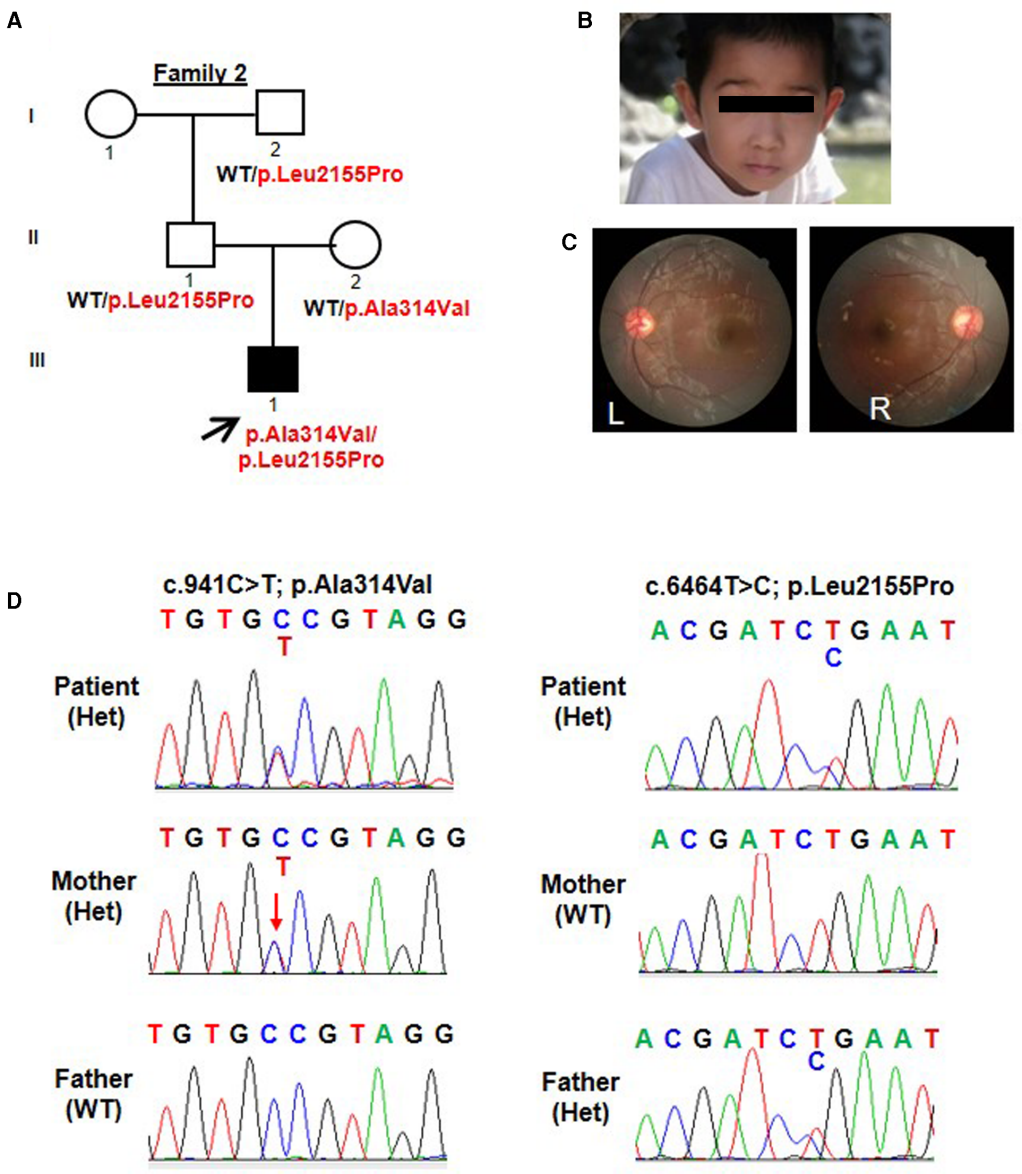
Trio-based WES identified compound heterozygous variants (p.Ala314Val; p.Leu2155Pro) in *TENM3* in a patient with speech delay and motor developmental delay. (**A**) Pedigree and genotype information on members of family 2. Squares indicate males, circles indicate females, filled symbols indicate affected individuals, and open symbols indicate healthy individuals. Patient 2 is denoted by a black arrow. The proband carried compound heterozygous variants: c.941C > T; p.Ala314Val and c.6464T > C; p.Leu2155Pro. (**B**) Facial picture of the proband at the age of 3 years old with resolved esotropia. His prominent and big ears were noted. (**C**) Fundus examination of patient 2 showed no structural anomalies. (**D**) Sequencing chromatograms of the compound heterozygous *TENM3* variants (c.941C > T; p.Ala314Val and c.6464T > C; p.Leu2155Pro) in patient 2 and the parents. WT, wild type; Het, heterozygous.

Patient 2 was the 3-year-and-5-month-old son of Chinese non-consanguineous parents. His weight was 17 kg (0.8 SD) and his height was 101 cm (0.2 SD). At birth, it was noted that he had bilateral esotropia. At the age of 3 years old, his bilateral esotropia was resolved (Figure 2B). What's more, his anterior segment and fundus examination showed no structural anomalies (Figure 2C). He had astigmatism in both eyes (−1.25 DC). He had mild motor delay. He was able to walk without support at 17 months. He had significant speech delay, not producing any meaningful words at 2 years and 5 months and speaking a few simple words (about 10 words) at 3 years and 5 months. He showed poor eye contact and was not interested in his surroundings, so his social interaction was abnormal. According to the Gesell Developmental Diagnostic Scale for children, the proband's delayed speech indicated a developmental age of only 18 months. He was also evaluated by the Autistic Behavior Checklist (ABC) and Childhood Autism Rating Scale (CARS) (ABC: score 40; CARS: score 32). His blood counts, liver and renal function tests, thyroid profile, and metabolic screen by mass spectrometry were normal. His hearing evaluation was also normal. His prominent and big ears were noted. A brain MRI did not show any intracranial abnormalities. His father had no abnormal eye appearance, but his eyes were myopic (−7.00 DS), and his mother was healthy. His grandfather had a history of fundus abnormality.

### Genetic analysis

Initial genetic testing for patient 1 was carried out *via* WES, revealing two heterozygous variants in the *TENM3* gene (Genbank association number: NM_001080477): c.3847_3855del; p.Leu1283_Ser1285del and c.3698_3699insA; p.Thr1233Thrfs*20 (Figure 1A). Sanger sequencing of the parents confirmed that the variant p.Leu1283_Ser1285del was inherited maternally. The variant p.Thr1233Thrfs*20 was confirmed to be *de novo*. The two variants were both located in exon 19. A TA clone sequencing including the fragment of exon 19 demonstrated that the two variants occurred biallelically (Figure 1D). The two variants were absent from the control database gnomAD. Based on the American College of Medical Genetics and Genomics (ACMG) guidelines, the variant c.3698_3699insA; p.Thr1233Thrfs*20 can be categorized as pathogenic (PVS1 + PM2 + PP4) and the variant c.3847_3855del; p.Leu1283_Ser1285del can be categorized as a variant of likely pathogenic (PM2 + PM3 + PM4).

Similarly, patient 2 had a WES that revealed compound heterozygous variants of the *TENM3* gene, c.941C > T; p.Ala314Val and c.6464T > C; p.Leu2155Pro (Figure 2A). Sanger sequencing confirmed that his mother carried the c.941C > T (p.Ala314Val) mutation and his father carried the c.6464T > C (p.Leu2155Pro) mutation (Figure 2D). The p.Ala314Val variant was absent from the gnomAD. The p.Leu2155Pro variant occurred once heterozygously in the gnomAD. Both the missense changes yielded predominantly deleterious prediction scores using five algorithms (Polyphen2_HDIV, MutationTaster, SIFT, Provean, and REVEL); the predicted results can be found in the Supplementary Material.

**Figure 3:**
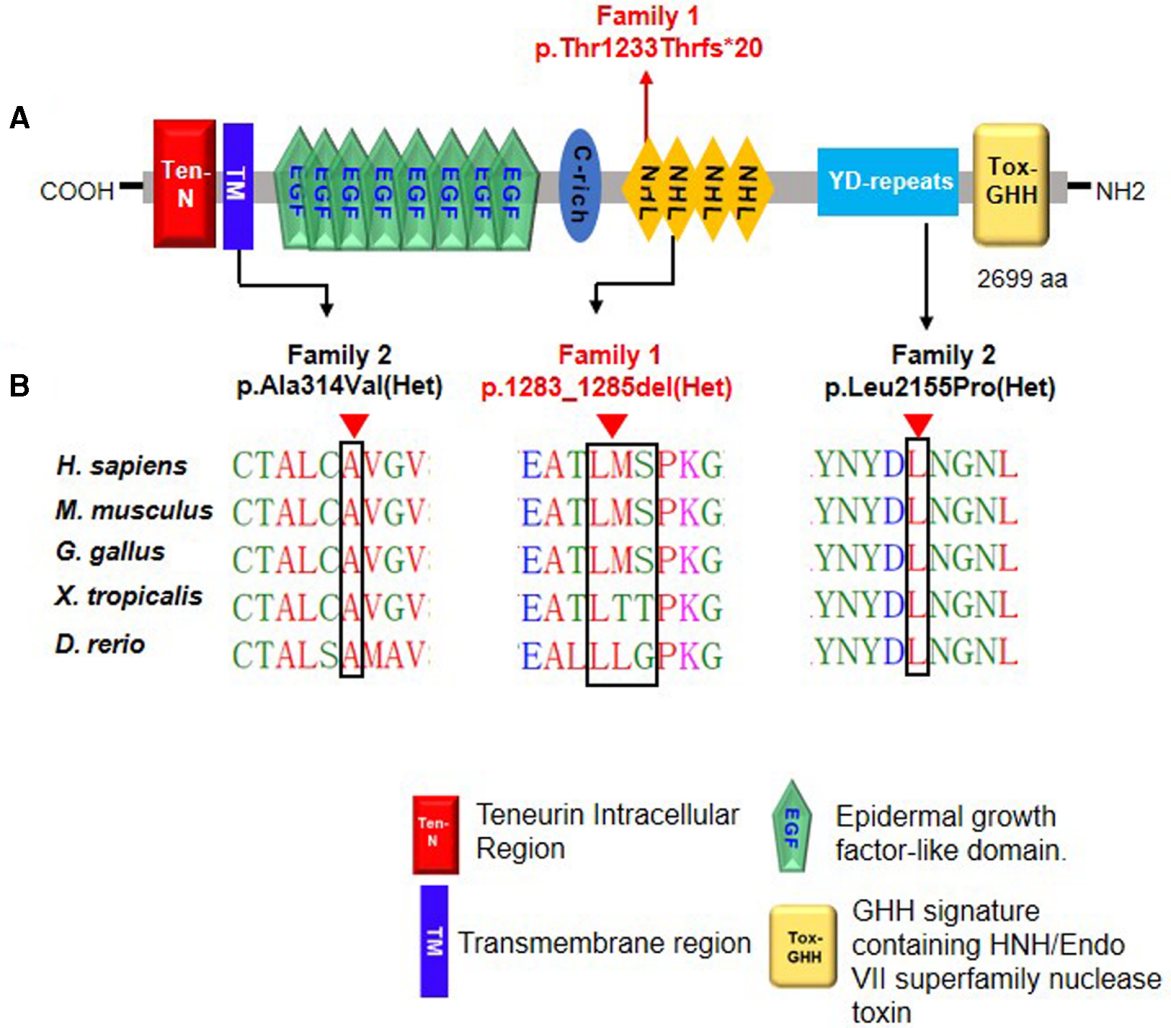
Schematic diagram of *TENM3* functional domains and *TENM3* variants identified in this study. (**A**) Depicts the protein domain structure of human TENM3 showing the domain position of the index heterozygous *TENM3* variants. aa, amino acids. (**B**) Evolutionary conservation of amino acid position Ala1283-Ser1285 and Leu2155 in TENM3 protein across evolution.

**Figure 4:**
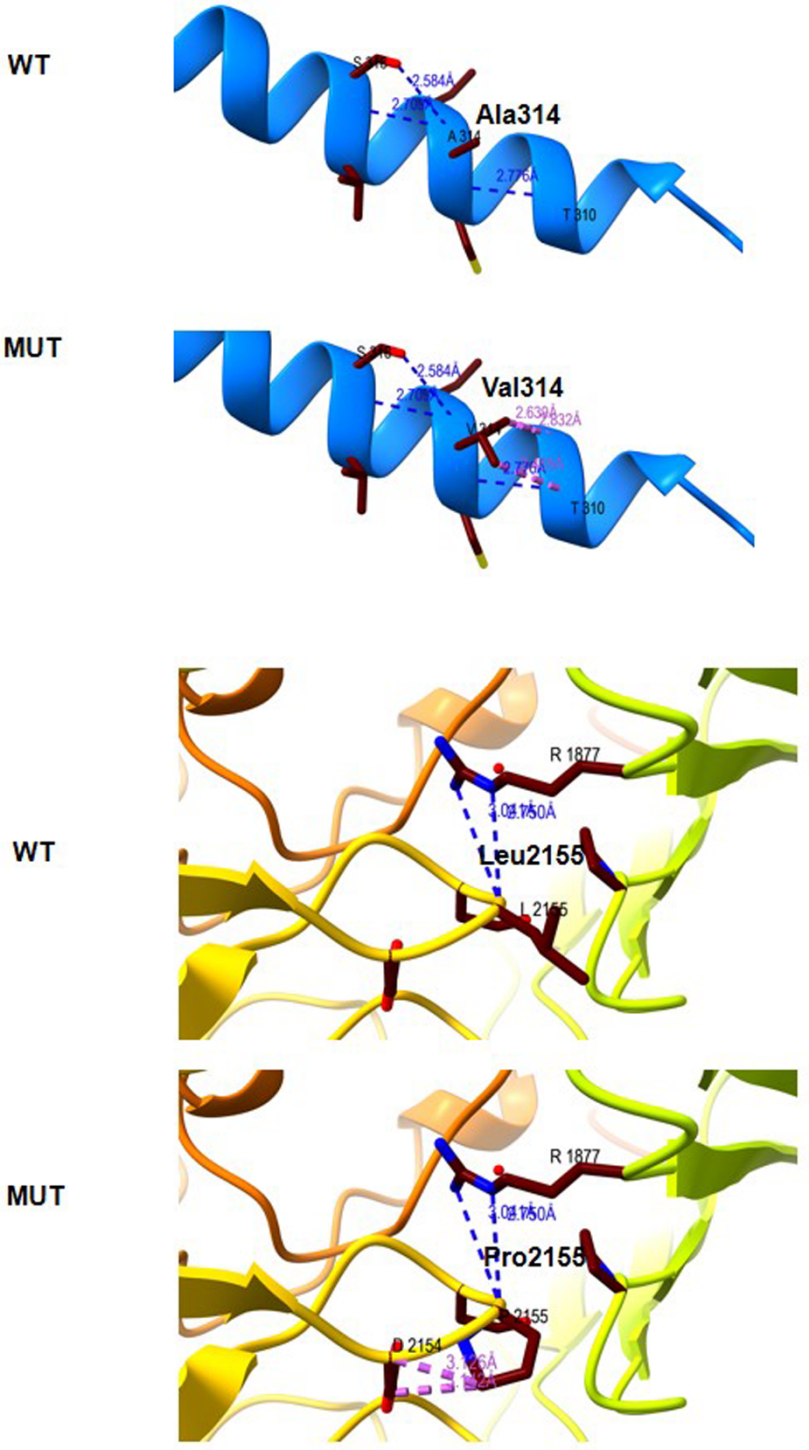
Molecular modeling of the wild type (WT) and mutant TENM3 protein (Mut). Three-dimensional structural modeling of the TENM3 protein showed that the mutations did not change the hydrogen bonding in protein (blue), but repulsive force (purple) was generated between the R group of amino acid side chain and other nearby groups.

The Ala314 change was located in the transmembrane domain and was evolutionarily well-conserved from *Homo sapiens* to *zebrafish* (Figures 3A,B). The Leu2155 residue was located in the YD-repeats domain and was well-conserved to zebrafish as well (Figures 3A,B). According to the ACMG guidelines, both variants, c.941C > T; p.Ala314Val and c.6464T > C; p.Leu2155Pro, can be categorized as variants of unknown significance (PM2 + PP2 + PP3). In evaluating the deleteriousness of the two missense variants in patient 2, three-dimensional structural modeling of the TENM3 protein showed that the mutations did not change the hydrogen bonding in the protein (blue), but repulsive force (purple) was generated between the R group of amino acid side chain and other nearby groups, which is unfavorable to the folding of the active protein and results in protein conformational instability (Figure 4).

## Discussion

To date, only eight patients with recessive *TENM3* variants have been described; information on the reported patients is shown in Table 1. The non-syndromic microphthalmia cases with recessive *TENM3* variants only presented with moderate or severe eye abnormalities, including microphthalmia, microcornea, and retinal and iris coloboma, while *TENM3* syndromic cases had additional abnormalities, such as craniofacial, renal, genital, cardiac, brain, and skeletal (9, 13, 14). To expand the *TENM3* gene-related phenotypic spectrum, we describe the clinical features of two Chinese patients with compound heterozygous variants in *TENM3*. The main characteristic feature of this syndrome is eye involvement (15). All previously reported patients presented with moderate or severe eye abnormalities, including colobomatous microphthalmia, ocular coloboma, and cataract (9, 13, 14). The ocular features of patient 1 in our study are highly consistent with previous reports. However, patient 2, who harbored two missense variants (c.941C > T; p.Ala314Val and c.6464T > C; p.Leu2155Pro), did not have a phenotype related to microphthalmia, microcornea, or iris coloboma. At birth, it was noted that he had bilateral esotropia. However, his bilateral esotropia was resolved at the age of 3 years old. Furthermore, his anterior segment and fundus examinations showed normal structure. A literature review revealed that all variants in previously reported cases with microphthalmia and/or coloboma with developmental delay had biallelic truncating variants. The only reported case with compound heterozygote missense likely pathogenic sequence variations in *TENM3* (p.Ala1349Gly and p.Arg2563Trp) showed right eye microphthalmia, sclerocornea of both eyes, anterior segment dysgenesis, and intellectual disability (14). We speculate that the missense mutations in patient 2 may have a mild effect on the structure of the TENM3 protein, which is associated with mild ocular symptoms. However, this must be further verified by *in vitro* experiments or animal experiments.

**Table 1:** Clinical manifestations of patients with variants in *TENM3*.

Clinical characteristics	Patient 1	Patient 2	Patient 3	Patient 4	Patient 5	Patient 6	Patient 7	Patient 8	Patient 9	Patient 10
Age	11	9	9	6	5y6m	4y3m	-	32	5m	3y5m
Sex	Male	Female	Male	Male	Female	Female	-	Male	Female	Male
Genotype	Homozygous c.2083dup; p.Thr695Asnfs*5	Homozygous c.2083dup; p.Thr695Asnfs*5	Homozygous c.2968-2A > T; p. Val990Cysfs*13	Compound heterozygous c.7687C > T; p. Arg2563Trp and c.4046C > G; p. Ala1349Gly	Homozygous c.1857T > A; p. Cys619*	Homozygous c.1857T > A; p. Cys619*	Homozygous c.1558C > T; p. (Arg520*)	Homozygous c.5069-1G > C; p.1690Asp > Glyfs*2	Compound heterozygous c.3698_3699insA; p.Thr1233Thrfs*20 and c.3847_3855del CTCATGAGT; p.Leu1283_Ser1285del	Compound heterozygous c.941C > T; p.Ala314Val and c.6464T > C; p.Leu2155Pro
Type of mutation	Frameshift	Frameshift	Splice	Missense	Nonsense	Nonsense	Nonsense	Frameshift	Frameshift/In-frame deletion	Missense
Motor development	Normal	Normal	Delayed	Delayed	Delayed	Delayed	-	Normal	Delayed	Delayed
Cognition	Normal	Normal	Delayed	Delayed	Delayed	Delayed	-	Delayed	Delayed	Delayed
Ptosis	No	No	No	No	Unilateral (left)	Bilateral partial ptosis	-	Yes	No	No
Microphthalmia	Yes	Yes	Yes	Yes	No	No	Yes	Yes	Yes	No
Micro cornea	Yes	Yes	Yes	Yes	Yes	Yes	-	-	Yes	No
Iris coloboma	Yes	Yes	Yes	-	Yes	Yes	Yes	-	No	No
Retinal coloboma	Yes	Yes	Yes	-	Yes	Yes	Yes	No	Yes	No
Visual acuity	20/50 (R) Hand movement (L)	20/200 (R) 20/300 (L)	Hand movement both eyes	-	6/36 both eyes	6/36 both eyes	-	-	-	Astigmatism in both eyes (−1.25 DC)
Facial dysmorphic features	-	-	Mild	-	-	-	-	-	Mild	-
References	Aldahmesh and others 2012	Aldahmesh and others 2012	Chassaing and others 2016	Singh and others 2019	Stephen and others 2018	Stephen and others 2018	Farrah Islam and others 2020	Gholami Yarahmadi and others 2022	Our present study	Our present study

* Means termination codon.

The mother of patient 1 with heterozygosity of the p.Thr1233Thrfs*20 variant had left exotropia and graduated from middle school with poor grades. The gnomAD constraint metric of TENM3 for loss of function is 1.0, indicating a high intolerance for heterozygous loss of function variants. However, no neurologic or ocular phenotypes were reported in individuals harboring a heterozygous allele in *TENM3*.

The two patients in our study had delayed developmental milestones similar to those observed in patients with recessive *TENM3* variants: these included global developmental delay, speech delay, and motor developmental delay (8). Brain MRIs showed no structural abnormalities. Notably, the presence of microcephaly in patient 1 potentially expands the neurologic phenotype associated with loss of function variants in *TENM3*, as microcephaly has not previously been described. The two patients received rehabilitation training in our department and their motor function and language skills both improved, but there was no improvement in their eye symptoms.

In conclusion, we reported the clinical features of two cases with recessive variants in *TENM3*. While the majority of *TENM3* syndromic or non-syndromic cases are truncating, missense variants have been described much less. It should be noted that biallelic missense variants in *TENM3* seem to have a minor impact on eye involvement.

## Data Availability

The data presented in the study are included in the article/**Supplementary Material**, further inquiries can be directed to the corresponding author.
